# Is Alcohol an “Essential Good” During COVID‐19? Yes, but Only as a Disinfectant!

**DOI:** 10.1111/acer.14417

**Published:** 2020-08-27

**Authors:** Maria Neufeld, Dirk W. Lachenmeier, Carina Ferreira‐Borges, Jürgen Rehm

**Affiliations:** ^1^ Institute of Clinical Psychology and Psychotherapy (MN, JR) Technische Universität Dresden Dresden Germany; ^2^ Institute for Mental Health Policy Research (MN, JR) Centre for Addiction and Mental Health Toronto Ontario Canada; ^3^ Chemisches und Veterinäruntersuchungsamt Karlsruhe (DWL) Karlsruhe Germany; ^4^ WHO European Office for Prevention and Control of Noncommunicable Diseases (CF‐B) Moscow Russia; ^5^ Dalla Lana School of Public Health (JR) University of Toronto (UofT) Toronto Ontario Canada; ^6^ Department of Psychiatry (JR) Faculty of Medicine UofT Toronto Ontario Canada; ^7^ Department of International Health Projects (JR) Institute for Leadership and Health Management I. M. Sechenov First Moscow State Medical University Moscow Russia

**Keywords:** Alcohol, Alcohol Surrogates, COVID‐19, Disinfectant, Infectious Disease, Novel Coronavirus, Poisoning, Public Health

Alcohol adversely affects people around the world on a large scale even in nonpandemic times, with about 3 million deaths attributed to alcohol use each year (Shield et al., 2020). During the current coronavirus disease (COVID‐19) pandemic, a variety of government reactions related to alcohol control was seen, with some countries banning the sale of alcohol outright, and others formally declaring off‐premises sales and alcohol delivery services to be “essential,” allowing for additional forms of delivery and weakened restrictions on its availability (Rehm et al., 2020; Reynolds and Wilkinson, 2020). Although sales bans, especially total bans, can be problematic, they do follow the public health rationale and the existing evidence that reducing the availability of retail alcohol will result in less consumption and, therefore, less alcohol‐related harm (Chisholm et al., 2018). Declaring beverage alcohol to be an “essential good” during a pandemic, therefore, seems to run counter to this and might signal the close relationship and influence that the alcohol industry may have on policy decision‐makers (Hamilton, 2020). However, there is one scenario in which alcohol could be considered essential during the COVID‐19 pandemic, namely the diversion of beverage alcohol to be used as a disinfectant in response to the increased demand for such products (e.g., hand sanitizers and household cleaning agents). In the following paragraphs, this idea is further explored against the backdrop of the alcohol policy response to COVID‐19, and health and safety implications are discussed.

Some outcomes caused or worsened by the consumption of alcohol, such as suicide or domestic violence, may increase due to the interaction of enforced social isolation, the disruption of usual work–life rhythms, and their associated distress (World Health Organization Regional Office for Europe, 2020a). Nevertheless, many false beliefs surround alcohol in connection with COVID‐19, especially regarding the alleged health benefits of alcohol. For example, mass methanol poisonings occurred in Iran, a country severely affected by the pandemic, and in which alcohol is illegal, following rumors that alcohol would ward off the virus (at least 5,000 poisonings and more than 700 deaths reported (Farmer, 2020)). In some Iranian provinces, the death toll due to alcohol poisonings was higher than that due to COVID‐19. Similar methanol poisonings occurred in Azerbaijan (Media.az., 2020) and Turkey (Regnum.ru., 2020) as consumers tried to protect themselves against the virus through ingestion of illegally sold alcohol. A recent opinion poll from Russia revealed that 90% of Russians have stockpiled alcohol at home because of the pandemic, with only 5% intending to use it as a hand sanitizer, and 69% believe that alcohol consumption helps protect against COVID‐19 (Ria.ru., 2020). To counter these dangerous assumptions, the WHO published materials stating that alcohol ingestion does not destroy SARS‐CoV‐2, but actually facilitates infection and worsens its course, as it is immunosuppressive (World Health Organization Regional Office for Europe, 2020a; World Health Organization Regional Office for Europe, 2020b).

During the pandemic, several countries (e.g., South Africa, Thailand, and India) have introduced total bans on alcohol sales, mainly aiming to minimize the risks of alcohol‐fueled domestic violence under lockdowns, reduce the burden to the healthcare system from alcohol‐related health emergencies, and prevent the virus from spreading further as intoxicated individuals might not practice physical distancing and personal hygiene (Nadkarni et al., 2020; Rehm et al., 2020). Other countries have introduced partial bans for similar reasons. For instance, Georgia has closed all liquor stores as part of an emergency response introduced in April (Legislative Herald of Georgia, 2020), and Greenland introduced temporary bans in some communities to protect children from adults’ drinking (George, 2020), while Russia limited sale hours in some regions to reduce alcohol‐attributable harms (Antonova, 2020). While the impact of these interventions has yet to be evaluated, interventions in other countries provoked some immediate reactions and consequences, highlighting the complexity of the issue. France, for instance, had to revoke its local sales ban within 24 hours to avoid triggering the side effects of withdrawal in people with alcohol dependence (The Local, 2020). In Mexico, where alcohol sales were prohibited in several regions, hundreds of poisonings from the ingestion of illegal and often methanol‐tainted alcohol have occurred since May, killing almost 200 people (Mexico News Daily, 2020).

In some, mostly high‐income, countries in North America and Europe, alcohol was declared—implicitly or explicitly—to be “essential.” For instance, alcohol retailers were included on the lists of “essential services” in Canada, New Zealand, the United States, and the United Kingdom, and were allowed to remain open during lockdown (Hamilton, 2020).

Although managed access to alcohol for people with alcohol dependence during the ongoing pandemic can be considered as an essential service as part of a harm reduction approach (Brar et al., 2020), this should not be used as an argument to increase availability of retailed alcohol for the general population. The reduced serving opportunities due to the shutdown of on‐premises facilities such as bars and restaurants might have been overcompensated for by at‐home drinking occasions, especially in countries where legislative changes were made to allow home delivery or online sales as in Canada, Latvia, and the United States (Latvian Public Broadcasting, 2020). It seems too early to evaluate the pandemic’s impact on sales and consumption, but preliminary data indicate sales increases of 14 to 28% in high‐income countries such as the United Kingdom and the United States in the first weeks of pandemic, possibly in part due to stockpiling purchases (Nadkarni et al., 2020). In some cases, these increases were steep—in Russia, a 30% increase in sales was observed in the first week of April despite considerable toughening of sales restrictions in some regions (Sergeeva and Krylova, 2020). The described changes—the loosening of alcohol availability regulations to allow for online sales and delivery services, based on the argument that easy availability of beverage alcohol is “essential”—likely led to increased consumption.

The one scenario in which alcohol could indeed be considered essential based on evidence is the diversion of beverage alcohol to be used for disinfecting purposes. Given supply shortages of food‐grade and pharmaceutical ethanol at the beginning of the pandemic, several countries permitted the temporary use of fuel‐ and technical‐grade alcohol, which contains more impurities than beverage alcohol (Deutsche Apothekenzeitung, 2020; Law Business Research, 2020). Shortly thereafter, safety concerns were raised over the high levels of carcinogens and other potentially harmful substances contained in some of these alcohols (Cable News Network, 2020). Moreover, several methanol poisonings due to the ingestion of hand sanitizers were observed in North America, leading the U.S. Food and Drug Administration to recall several products (Federal Drug Administration (FDA), 2020).

Given these recent developments, repurposing not only alcohol production facilities for the production of hand sanitizers, but also turning alcoholic beverages into disinfectants, seems to be a viable option to respond to the current needs. For instance, a recent incident in Poland described how confiscated illegal vodka was used as a disinfectant rather than being destroyed (The Brussels Times, 2020), and another report suggested that European vineyards could turn a billion liters of wine into disinfectants in a “crisis distillation” program (Dailymail.co.uk, 2020).

Although only solutions containing 60% alcohol are typically recommended for disinfection, recent evidence suggests that ethanol and isopropanol efficiently inactivate SARS‐CoV‐2 in 30 seconds at a concentration of >30%. Therefore, commercial spirits (about 40% alcohol) could be suitable for hand or surface disinfection if no other disinfectant were available, which corresponds with anecdotal evidence that vodka was typically used for disinfection in Soviet countries in times of economic crisis and the resulting shortages of medical supplies.

However, clear messages on the safe use and storage of alcohol‐based disinfectants are needed because substantial increases in poisonings due to the ingestion of rubbing alcohol and household cleaning products were recorded in some countries, including hand sanitizer exposures in children (PR Newswire, 2020) and fatal poisonings with methanol‐based hand sanitizer (The New York Times Company, 2020). The misuse of hand sanitizers and other alcoholic liquids not intended for consumption is a known, but still under‐researched phenomenon, which is mostly observed in marginalized individuals with alcohol use disorders, and factors like affordability and physical availability of these products, especially in times of crisis, play a key role in their consumption (Elton‐Marshall et al., 2020; Lachenmeier et al., 2007; Neufeld et al., 2019). In the context of COVID‐19, shifts to surrogate alcohol were observed in some countries, possibly as an unintended consequence of alcohol sales bans (Mexico), but also following misinformation that alcohol protects against the virus (Azerbaijan). Due to the resulting increase in demand for disinfectants, however, the opposite scenario could become the norm: Cheap vodka and other spirits (>40% alcohol) might be diverted for disinfecting purposes.

While misuse of surrogate alcohol should be avoided at all costs, both during the COVID‐19 global crisis and once it is over, the “diversion” of alcoholic beverages to other life‐saving uses is clearly warranted during the current pandemic.

## Acknowledgement

Open access funding enabled and organized by Projekt DEAL.

## Conflict of Interest

The authors declare that they have no conflict of interests.

## Disclaimer

Carina Ferreira‐Borges is a staff member of the World Health Organization. The authors alone are responsible for the views expressed in this publication, and they do not necessarily represent the decisions or the stated policy of the World Health Organization.

References

Antonova
V
 (2020) Skupili ves' «Bojaryshnik»: kak izmenilis' alkogol'nye predpochtenija rossijan na samoizoljacii. [They bought all the “Hawthorn”: how the alcoholic preferences of Russians on self‐isolation changed]. [Online]. Available from: https://radiokp.ru/obschestvo/skupili‐ves‐boyaryshnik‐kak‐izmenilis‐alkogolnye‐predpochteniya‐rossiyan‐na‐samoizolyacii_nid26981_au8073au. Accessed July 10, 2020.

Brar
R
, 
Bruneau
J
, 
Butt
P
, 
Goyer
ME
, 
Lim
R
, 
Poulin
G
, 
Sereda
A
, 
Robinson
S
, 
Ross
J
, 
Wood
E
 (2020) Medications and Other Clinical Approaches to Support Physical Distancing for People Who Use Substances During the COVID‐19 Pandemic: National Rapid Guidance Document. Canadian Research Initiative in Substance Misuse, Vancouver, BC.
Cable News Network
(2020) 
*A plan to ease the hand sanitizer shortage could go bust* [Online]. Available from: https://edition.cnn.com/2020/05/01/business/hand‐sanitizer‐ethanol‐fda/index.html. Accessed July 07, 2020.

Chisholm
D
, 
Moro
D
, 
Bertram
M
, 
Pretorius
C
, 
Gmel
G
, 
Shield
K
, 
Rehm
J
 (2018) Are the “best buys” for alcohol control still valid? An update on the comparative cost‐effectiveness of alcohol control strategies at the global level. J Stud Alcohol Drugs
79:514–522.30079865
Dailymail.co.uk
(2020) European vineyards may have to turn a BILLION litres of wine into industrial alcohol amid a huge surplus caused by the coronavirus outbreak, French politician warns. [Online]. Available from: https://www.dailymail.co.uk/news/article‐8264889/European‐vineyards‐turn‐BILLION‐litres‐wine‐industrial‐alcohol.html?ito=rss‐flipboard. Accessed July 13, 2020.
DEUTSCHE APOTHEKENZEITUNG
(2020) Brennereien und Zuckerfabriken versorgen Apotheken mit Alkohol [Distilleries and sugar factories supply pharmacies with alcohol]. [Online]. Available from: https://www.deutsche‐apotheker‐zeitung.de/news/artikel/2020/03/26/brennereien‐und‐zuckerfabriken‐versorgen‐apotheken‐mit‐alkohol. Accessed July 15, 2020.

Elton‐Marshall
T
, 
Ali
F
, 
Hyshka
E
, 
Shahin
R
, 
Hopkins
S
, 
Imtiaz
S
, 
Graham
B
, 
Rehm
J
 (2020) Harm Reduction Worker Safety During the COVID‐19 Global Pandemic: National Rapid Guidance. Ontario, Canadian Research Initiative in Substance Misuse, Toronto, ON.

Farmer
B
 (2020) Toxic alcohol kills more than 700 in Iran following false reports it wards off coronavirus. [Online]. Available from: https://www.telegraph.co.uk/global‐health/science‐and‐disease/toxic‐alcohol‐kills‐700‐iran‐following‐false‐reports‐ward‐coronavirus/. Accessed May 19, 2020.
Federal Drug Administration (FDA)
(2020) FDA Updates on Hand Sanitizers with Methanol [Online]. Available from: https://www.fda.gov/drugs/drug‐safety‐andavailability/fda‐updates‐hand‐sanitizers‐methanol. Accessed July 10, 2020.

George
J
(2020) Greenland temporarily bans alcohol sales in some communities during coronavirus lockdown [Online]. Arctic Today. Available from: https://www.arctictoday.com/greenland‐temporarily‐bans‐alcohol‐sales‐in‐some‐communities‐during‐coronavirus‐lockdown/. Accessed July 10, 2020.

Hamilton
I
 (2020) Is alcohol really “essential” during covid‐19? [Online]. BMJ Opinion. Available from: https://blogs.bmj.com/bmj/2020/04/07/ian‐hamilton‐is‐alcohol‐really‐essential‐during‐covid‐19/. Accessed July 10, 2020.

Lachenmeier
DW
, 
Rehm
J
, 
Gmel
G
 (2007) Surrogate alcohol: what do we know and where do we go?
Alcohol Clin Exp Res
31:1613–1624.1768103410.1111/j.1530-0277.2007.00474.x
Latvian Public Broadcasting
(2020) Online alcohol sales allowed during Covid‐19 pandemic in Latvia. [Online]. Available from: https://eng.lsm.lv/article/economy/business/online‐alcohol‐sales‐allowed‐during‐covid‐19‐pandemic‐in‐latvia.a352700/. Accessed July 01, 2020.
Law Business Research
(2020) FDA revises guidance on using ethanol in hand sanitizer products during the COVID‐19 pandemic [Online]. Available from: https://www.lexology.com/library/detail.aspx?g=9e51dd89‐b8dc‐4e47‐b3dd‐e325ea6a1458. Accessed July 10, 2020.
Legislative Herald Of Georgia
(2020) "Sakartveloshi akhali k'oronavirusis gavrtselebis aghk'vetis miznit gasat'arebeli ghonisdziebebis damt'k'itsebis shesakheb“ sakartvelos mtavrobis 2020 ts'lis 23 mart'is No 181 dadgenilebashi tsvlilebis shet'anis taobaze. [On Approval of the Measures to be Taken to Prevent the Spread of the New Coronavirus in Georgia Amending the Resolution No 181 of the Government of Georgia of March 23, 2020].
Media.Az
(2020) Pochemu v period karantina ljudi bol'she otravljajutsja spirtom? Media.Az pogovorila s glavnym toksikologom strany. [Why are people more poisoned by alcohol during the quarantine period? Media.Az spoke with the country's chief toxicologist]. [Online]. Available from: https://media.az/society/1067776187/pod‐vliyaniem‐intoksikacii‐chelovek‐mozhet‐prosnutsya‐slepym‐toksikolog‐rasskazal‐o‐kolichestve‐otravleniy/ Accessed May 19, 2020.
Mexico News Daily
(2020) Adulterated liquor has killed 189 people since May 1. [Online]. Available from: https://mexiconewsdaily.com/news/adulterated‐liquor‐has‐killed‐189‐people‐since‐may‐1/. Accessed July 10, 2020.

Nadkarni
A
, 
Kapoor
A
, 
Pathare
S
 (2020) COVID‐19 and forced alcohol abstinence in India: the dilemmas around ethics and rights. Int J Law Psychiatry
71:101579.3276811310.1016/j.ijlp.2020.101579PMC7237931

Neufeld
M
, 
Wittchen
H‐U
, 
Ross
LE
, 
Ferreira‐Borges
C
, 
Rehm
J
 (2019) Perception of alcohol policies by consumers of unrecorded alcohol‐an exploratory qualitative interview study with patients of alcohol treatment facilities in Russia. Subst Abuse Treat Prev Policy
14:53.3175292610.1186/s13011-019-0234-1PMC6869256

Newswire
PR
 (2020) Washington Poison Center releases data on increased poison exposures during COVID‐19. [Online]. Available from: https://www.prnewswire.com/news‐releases/washington‐poison‐center‐releases‐data‐on‐increased‐poison‐exposures‐during‐covid‐19‐301062188.html. Accessed July 13, 2020.
Regnum.ru
(2020) V Turcii ot otravlenija alkogolem umerli 37 migrantov iz Turkmenii. [In Turkey, 37 migrants from Turkmenistan died from alcohol poisoning}. [Online]. Available from: https://regnum.ru/news/2892984.html. Accessed May 19, 2020.

Rehm
J
, 
Kilian
C
, 
Ferreira‐Borges
C
, 
Jernigan
D
, 
Monteiro
M
, 
Parry
CDH
, 
Sanchez
ZM
, 
Manthey
J
 (2020) Alcohol use in times of the COVID 19. Implications for monitoring and policy. Drug Alcohol Rev
39:301–304
3235888410.1111/dar.13074PMC7267161

Reynolds
J
, 
Wilkinson
C
 (2020) Accessibility of 'essential' alcohol in the time of COVID‐19: Casting light on the blind spots of licensing?
Drug Alcohol Rev
39:305–308.3232954810.1111/dar.13076
RIA.RU
(2020) Glava "Trezvoj Rossii": rossijane pokupajut alkogol' iz‐za COVID‐19 [Head of “Sober Russia”: Russians buy alcohol because of COVID‐19]. [Online]. Available from: https://ria.ru/20200331/1569378042.html. Accessed April 23, 2020.

Sergeeva
P
, 
Krylova
E
 (2020) Operativnaja ocenka potrebitel'skoj aktivnosti rossijan 30 marta – 5 aprelja [Operational assessment of Russians' consumer activity on March 30 ‐ April 5] [Online]. Available from: https://www.sberbank.ru/common/img/uploaded/files/pdf/analytics/pa3003‐0504.pdf.

Shield
K
, 
Manthey
J
, 
Rylett
M
, 
Probst
C
, 
Wettlaufer
A
, 
Parry
CDH
, 
Rehm
J
 (2020) National, regional, and global burdens of disease from 2000 to 2016 attributable to alcohol use: a comparative risk assessment study. Lancet Public Health
5:E51–E61.3191098010.1016/S2468-2667(19)30231-2
The Brussels Times
(2020) (April 20). Coronavirus: Poland used confiscated vodka as disinfectant [Online]. The Brussels Times, Brussels. Available from: https://www.brusselstimes.com/all‐news/101752/coronavirus‐poland‐to‐use‐confiscated‐vodka‐alcohol‐as‐disinfectant/. Accessed April 21, 2020.
The Local
(2020) UPDATE: Halt on French local authority's alcohol ban during lockdown [Online]. Available from: https://www.thelocal.fr/20200324/french‐local‐authority‐bans‐sale‐of‐alcohol‐during‐lockdown. Accessed July 10, 2020.
The New York Times Company
(2020) 3 Die in New Mexico After Drinking Hand Sanitizer, Officials Say [Online]. Available from: https://www.nytimes.com/2020/06/26/us/3‐dead‐drinking‐hand‐sanitizer.html. Accessed July 15, 2020.
World Health Organization Regional Office for Europe
(2020a). Fact sheet ‐ Alcohol and COVID‐19: what you need to know. [Online]. WHO Regional Office for Europe, Copenhagen, Denmark. Available from: http://www.euro.who.int/en/media‐centre/sections/fact‐sheets/2020/fact‐sheet‐alcohol‐and‐covid‐19‐what‐you‐need‐to‐know. Accessed April 21, 2020.
World Health Organization Regional Office for Europe
(2020b) Frequently asked questions (FAQ) about alcohol and COVID‐19. [Online]. WHO Regional Office for Europe, Copenhagen, Denmark. Available from: https://www.euro.who.int/__data/assets/pdf_file/0007/442690/FAQ‐COVID‐19‐alcohol.pdf. Accessed April 21, 2020.
